# Magnetic patterning: local manipulation of the intergranular exchange coupling via grain boundary engineering

**DOI:** 10.1038/srep11904

**Published:** 2015-07-09

**Authors:** Kuo-Feng Huang, Jung-Wei Liao, Cheng-Yu Hsieh, Liang-Wei Wang, Yen-Chun Huang, Wei-Chih Wen, Mu-Tung Chang, Shen-Chuan Lo, Jun Yuan, Hsiu-Hau Lin, Chih-Huang Lai

**Affiliations:** 1 Department of Materials Science and Engineering, National Tsing Hua University, Hsinchu, 300, Taiwan; 2 Material and Chemical Research Laboratories, Nanotechnology Research Center, Industrial Technology Research Institute, Hsinchu, 310, Taiwan; 3 Department of Physics, University of York, Heslington, York, YO10 5DD, United Kingdom; 4 Department of Physics, National Tsing Hua University, Hsinchu, 300, Taiwan

## Abstract

Magnetic patterning, with designed spatial profile of the desired magnetic properties, has been a rising challenge for developing magnetic devices at nanoscale. Most existing methods rely on locally modifying magnetic anisotropy energy or saturation magnetization, and thus post stringent constraints on the adaptability in diverse applications. We propose an alternative route for magnetic patterning: by manipulating the local intergranular exchange coupling to tune lateral magnetic properties. As demonstration, the grain boundary structure of Co/Pt multilayers is engineered by thermal treatment, where the stress state of the multilayers and thus the intergranular exchange coupling can be modified. With Ag passivation layers on top of the Co/Pt multilayers, we can hinder the stress relaxation and grain boundary modification. Combining the pre-patterned Ag passivation layer with thermal treatment, we can design spatial variations of the magnetic properties by tuning the intergranular exchange coupling, which diversifies the magnetic patterning process and extends its feasibility for varieties of new devices.

Information technology makes great use of various magnetic devices including magnetic patterned media^1^, magnetic random access memory^2^, magnetic domain wall devices^3^,^4^, and spin-wave devices^5^. A common approach to fabricate these nanostructure devices is the conventional lithography technique^6^,^7^ – excess regions are physically removed to define the desired spatial patterns with uniform magnetic properties. On the other hand, if the magnetic properties can be spatially controlled without physical etching, it can open up a new route for device designs with novel functionality. The so-called magnetic patterning is proposed to create the spatial distribution of magnetic properties by rearranging interface^1^, inducing order-disorder phase transformation^8^,^9^,^10^, doping additional elements^11^, or generating chemical reactions^12^,^13^. The magnetic patterning is now mainly realized by ion implantation^14^, and several unique devices have been demonstrated, such as the one-way domain wall motion shift registers^15^ or magnonic crystals^16^. Other magnetic patterning methods with different working mechanisms, including nano-indentation^17^, local thermal treatment^18^, or local anisotropy modification by adjacent layers^19^,^20^, have also been proposed. Most of the methods share the similar strategy by altering the spatial distribution of the effective magnetic anisotropy (K_eff_), sometimes accompanying the change of saturation magnetization (M_s_) as in ion implantation. To escape from the constraints imposed by changing magnetic anisotropy and/or saturation magnetization, is it possible to find another tuning knob to realize magnetic patterning for device designs?

In addition to modifying K_eff_ and M_s_, controlling microstructure is another way to modify magnetic properties, especially with regard to grain boundaries which has been widely investigated in recording media^21^ and permanent magnets^22^. By adding intergranular additives, one can engineer grain boundaries in recording media and permanent magnets for magnetic hardening, which may accompany modified intergranular exchange coupling. However, these approaches usually alter grain boundary structure uniformly in the entire sample and no spatial manipulation can be achieved.

In this work, we demonstrate a different approach for magnetic patterning by locally engineering the grain boundaries with altered intergranular exchange coupling. With pre-patterned passivation layers and thermal treatments, we demonstrate the magnetic patterning on Co/Pt multilayers (MLs), which are used as the model system due to its easy fabrication and perpendicular anisotropy for potential applications^23^. The magnetic analyses and microstructure investigations are carried out to reveal the working principles for this magnetic patterning method. With rapid thermal annealing (RTA) process, we found that stress relaxation in Co/Pt MLs brings about modification of grain boundary structures and the corresponding magnetic properties. Meanwhile, by adopting pre-patterned passivation layers, we can spatially control the degree of stress relaxation in the designated regions with lateral modulation of the nucleation field (H_n_), the domain wall propagating field (H_p_), and the domain structures in Co/Pt MLs. In addition, we find that the same magnetic patterning can be achieved by Joule heating. By applying electric current, the magnetic properties in the specific regions can be altered. Different from the reported magnetic patterning methods, our unique grain boundary engineering approach alters local magnetic properties leaving both K_eff_ and M_s_ intact. Our proposed approach opens up a new avenue for magnetic patterning and can be easily incorporated into the existing process for device fabrication, for example, the BEOL (back-end-of-line) process, how the magnetic devices are usually integrated with other components^24^,^25^.

## Results and Discussions

### Magnetic patterning processes

**Figure 1:**
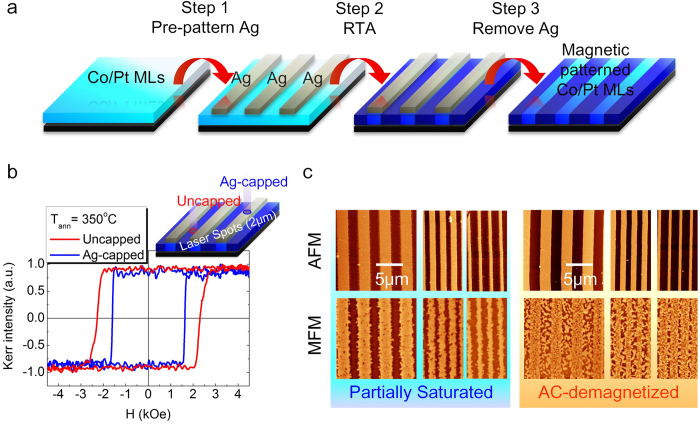
Demonstration on the proposed magnetic patterning method. (**a**) A flowchart of the proposed magnetic patterning process. (**b**) Hysteresis loops of the magnetic patterned Co/Pt MLs acquired from different regions by FMOKE. (**c**) AFM (upper row) and corresponding MFM images (lower row) of magnetic patterned Co/Pt MLs. The left (right) shows the partially saturated (ac-demagnetized) state with different width ratios of Ag-capped to uncapped stripes.

The flowchart of the proposed method for magnetic pattering is shown in Fig. 1(a). First, 20 nm thick Ag lines with different line width are pre-patterned on Co/Pt MLs by e-beam lithography and lift-off. Before RTA, the whole sample shows uniform magnetic properties. The sample is then annealed by using RTA in vacuum (5 × 10^−5^ Torr) with duration of 20 seconds. Regions with or without Ag capping show different responses to RTA, leading to heterogeneous distributions of the magnetic properties in different spatial regions. After RTA, the remaining Ag patterns can be selectively etched away by ammonia-based etching solution (NH_4_OH:H_2_O_2_ = 1:1) without damages to the magnetic properties of Co/Pt MLs (see supplementary Figure 1), yielding a flat magnetic film with spatially manipulated magnetic properties.

The hysteresis loops of the magnetic patterned Co/Pt MLs after 350 °C annealing is measured by focused magneto-optical Kerr effect (FMOKE) with a 2 μm probing spot in diameter, as shown in Fig. 1(b). Both loops probed in regions with or without the Ag capping layer, denoted as Ag-capped and uncapped respectively, show sharp magnetization transitions. However, the magnetic field (~2000 Oe) for magnetization switching in the uncapped regions is larger than that (~1500 Oe) in the Ag-capped regions.

The atomic force microscopy (AFM) and the magnetic force microscopy (MFM) images of the patterned Co/Pt MLs are taken before the removal of the Ag capping as shown in Fig. 1(c). In the left panel of Fig. 1(c), the AFM and MFM images respectively show the surface topography and magnetization distribution of Co/Pt MLs, after MLs are partially saturated, that is, an applied magnetic field with the magnitude between the switching field of uncapped and Ag-capped regions is applied on MLs before MFM images are taken. The AFM and MFM images clearly reveal that the regions with bright contrast in AFM images, corresponding to Ag-capped regions, possess reversed magnetization, showing bright contrast in MFM images; the regions with dark contrast correspond to the uncapped regions without magnetization reversal. On the other hand, the AFM and MFM images of the ac-demagnetized state are shown in the right two panels of Fig. 1(c). The regions with or without the Ag capping layer reveal quite different domain sizes: the Ag-capped regions show the larger domain sizes, but the uncapped regions show smaller ones. Our results clearly demonstrate that local manipulation of the magnetic properties and the ac-demagnetized domain structure in Co/Pt MLs can be realized by combining Ag pre-patterns and the RTA process.

### Working principles of the magnetic patterning: magnetic analyses

**Figure 2:**
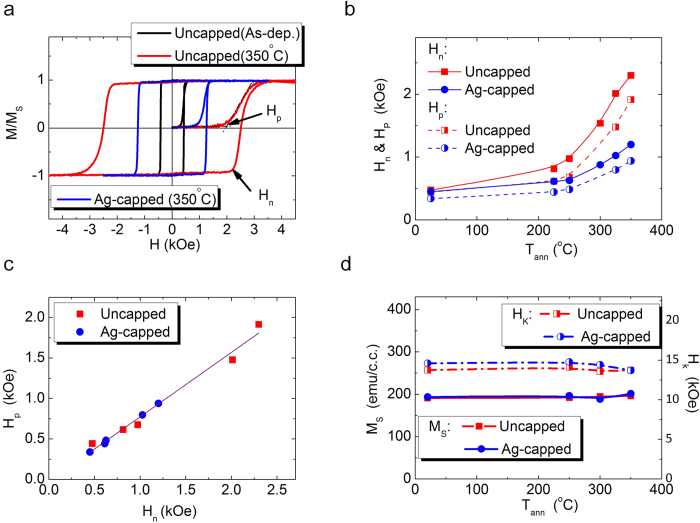
Sheet film properties after RTA process. (**a**) Typical out-of-plane hysteresis loops and its initial curves of Co/Pt MLs with different RTA temperatures (T_ann_) and capping conditions. The examples for defining H_n_ and H_p_ are also shown here. (**b**) Evolutions of H_n_ and H_p_ with increasing T_ann_. (**c**) Dependence between H_p_ and H_n_. (**d**) Evolutions of M_S_ and H_K_ with different T_ann_ and capping conditions.

Because the key ingredients for our proposed magnetic patterning are RTA and Ag capping layers, to understand the underlying mechanism, we first evaluate the modifications of magnetic properties on Co/Pt MLs with varied RTA temperatures and capping conditions. The representative out-of-plane hysteresis loops and initial curves of Co/Pt MLs with or without 20 nm Ag capping layer are shown in Fig. 2(a). All hysteresis loops of as-deposited and RTA samples reveal sharp magnetization transition, also observed in the magnetically patterned Co/Pt MLs shown in Fig. 1(b). It implies that the magnetization reversal for both sheet and patterned Co/Pt films is dominated by nucleation of reversed domains, followed by rapid domain wall expansion^26^. Based on the hysteresis loops and initial curves, we can extract the nucleation field (H_n_, the field beyond which the first magnetization reversal occurs from the saturation state) and the domain wall propagating field (H_p_, the field required for the onset of domain wall depinning). Both fields increase with elevating RTA temperature (T_ann_) below 350 ^o^C, as plotted in Fig. 2(b) (see Supplementary Figure 2 for the details when T_ann_ is above 350 ^o^C). We also observe that H_p_ is always smaller than the corresponding H_n_ and the H_p_/H_n_ ratios for all samples remain constant, as shown in Fig. 2(c). It is worth mentioning that the samples capped with Ag layers reveal smaller H_n_ and H_p_ than those without Ag under the same RTA condition, consistent with the case found in the corresponding regions in the Ag-patterned samples.

The magnetization reversal behavior of Co/Pt MLs, including H_n_ and H_p_, is reported to be determined by the interfacial anisotropy (K_s_) originating from Co/Pt interfaces^23^. The effective magnetic anisotropy (K_eff_) of Co/Pt MLs with perpendicular magnetic anisotropy can be expressed by


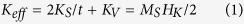


where t is the thickness of each Co layer, K_V_ the bulk anisotropy, and H_K_ the anisotropy field. After annealing, K_eff_ of Co/Pt MLs has been reported either to be reduced due to intermixing with lower K_s_^27^ or enhanced by interfacial alloying of ordered CoPt with higher K_V_^28^. Therefore, both H_n_ and H_p_ can change when K_eff_ is altered. However, as shown in Fig. 2(d), both the H_k_ and M_S_ are independent of T_ann_ here. It suggests that the significant enhancement of H_n_ and H_p_ after RTA cannot be simply explained by the modification of K_eff_ – a different mechanism is in order to explain the observed variations of H_n_ and H_p_.

**Figure 3:**
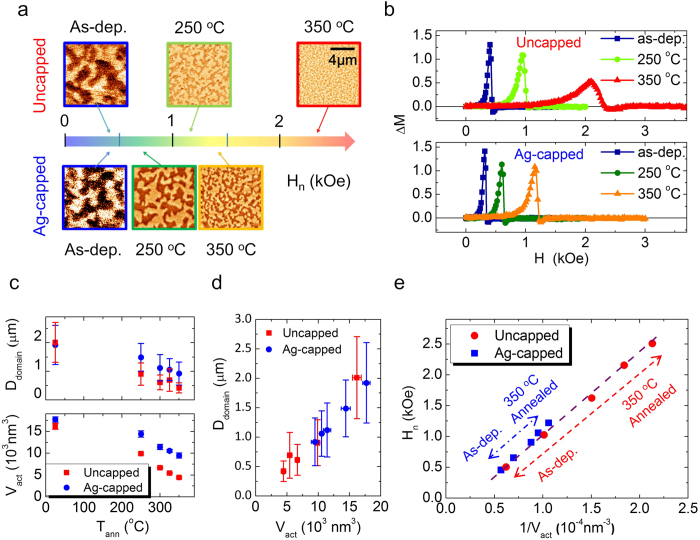
Magnetic analysis on the magnetic patterned Co/Pt MLs. (**a**) ac-demagnetized MFM images of the uncapped (upper row) and the Ag-capped samples (lower row) at the as-deposited state, and after 250 ^o^C, and 350 ^o^C RTA. (**b**) ΔM curves with different T_ann_ and capping conditions. (**c**) T_ann_ dependence of the ac-demagnetized domain size (D_domain_) and activation volume (V_act_). (**d**) Dependence between D_domain_ and V_act._ (**e**) Correlation between the reciprocal of activation volume (1/V_act_) and nucleation field (H_n_) of samples with different T_ann_. The red and blue arrows indicate the different amount of activation volume change from the as-deposited to the 350 ^o^C annealed for uncapped and Ag-capped cases, respectively.

In addition to the changes of H_n_ and H_p_, we also observed that sizes of the ac-demagnetized domains (D_domain_) are shrunk by elevating T_ann_, as shown in Fig. 3(a). Furthermore, the Ag-capped samples show the less modification of D_domain_ compared to the uncapped ones. Based on the micromagnetic simulations reported by R. H. Victora *et al.*^29^, with K_eff_ and M_s_ almost constant, the ac-demagnetized domain size of Co/Pt MLs can be determined by intergranular exchange coupling, which can be evaluated by using the ΔM curves^30^. In Fig. 3(b), the upper and lower panels show the ΔM curves of the uncapped and the Ag-capped samples, respectively. All samples exhibit positive ΔM peaks, indicating an exchange interaction dominated magnetization reversal^31^. The higher positive maximum intensity of the ΔM curve (ΔM(H)_Max_) indicates the stronger intergranular exchange coupling^32^. For all samples, the intergranular exchange coupling reduces with the elevating T_ann_ as the ΔM(H)_Max_ drops. The Ag-capped samples show a smaller drop of ΔM(H)_Max_ with T_ann_ than that of the uncapped counterparts, implying that the extra Ag capping layer hinders the reduction of intergranular exchange coupling during the RTA process. Furthermore, we also notice that the samples with smaller intergranular exchange coupling (lower ΔM(H)_Max_ and smaller D_domain_) shows higher H_n_, indicating that the intergranular exchange coupling should be an important factor for the magnetization reversal of Co/Pt MLs here.

To quantitatively evaluate the influence of intergranular exchange coupling on magnetization reversal, we checked the effective activation volume (V_act_) of each sample and used it as an indicator for the strength of intergranular exchange coupling. Note that V_act_ represents the size of an effective magnetic volume composed of physical grains coupled together through intergranular exchange coupling^33^,^34^. The effective activation V_act_ can be obtained by extracting the characteristic half time (t_1/2_) from magnetization relaxation curves of different applied magnetic fields (H_app_) and fitting curves into the equation shown below^26^,


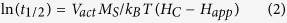


where k_B_ is the Boltzmann constant, T is measurement temperature (ambient temperature in this case), and H_c_ is the coercivity, respectively. As shown in Fig. 3(c), both V_act_ and D_domain_ decrease with increasing T_ann_, indicating that the RTA process reduces intergranular exchange coupling. Besides, because the D_domain_ and V_act_ are both affected by intergranular exchange coupling^33^,^34^, the linear dependence between V_act_ and D_domain_ revealed in Fig. 3(d) finds its natural explanation. Moreover, we found that the H_n_ is proportional to the reciprocal of the activation volume (1/V_act_), as plotted in Fig. 3(e), showing the increase of H_n_ accompanied by the reduction of V_act_. This relationship can be explained by the curling mode for nucleation^35^. In the curling mode, nucleation occurs via forming a curled spin structure with a lower energy barrier compared to that of nucleation via the Stoner-Wohlfarth model with coherent rotation. The nucleation energy barrier of the curling mode increases with shrinking V_act_ because it costs more exchange energy to form the curled spin structure. Therefore, the smaller V_act_ is, the higher H_n_ becomes (see Supplementary Note 1 for details). Noticeably, although the changes of 1/V_act_ for the Ag-capped and the uncapped samples are different, indicated by the length of the blue and red dash lines in Fig. 3(e) respectively, all data points of these two types of samples follow the same linear dependence. It indicates that H_n_ is directly determines by V_act_ of the Co/Pt MLs. The role of the Ag capping layer is mainly to control the size of V_act_, that is, the intergranular exchange coupling.

Now, we would like to discuss the effect of the intergranular exchange coupling on H_p_. When intergranular exchange coupling is lowered at grain boundaries, grain boundaries behave effectively as domain wall pinning sites so domain wall pinning strength can be enhanced^36^. Therefore, reduction of the intergranular exchange coupling leads to a smaller V_act_ as well as a higher domain wall pinning site density (n_pin_) simultaneously. The parameter 1/V_act_, a function of intergranular exchange coupling here, can also be used as an indicator for n_pin_. As the model proposed by V. Zablotskii *et al.*^37^, higher n_pin_ leads to higher H_p_. Hence, H_p_ increases with increasing 1/V_act_ and decreasing intergranular exchange coupling, as H_n_ does. Consequently, the direct dependence of H_p_ and H_n_ on the intergranular exchange coupling explains the correlation between H_p_ and H_n_ shown in Fig. 2(c).

### Working principles of the magnetic patterning: structural and microstructural analyses

**Figure 4:**
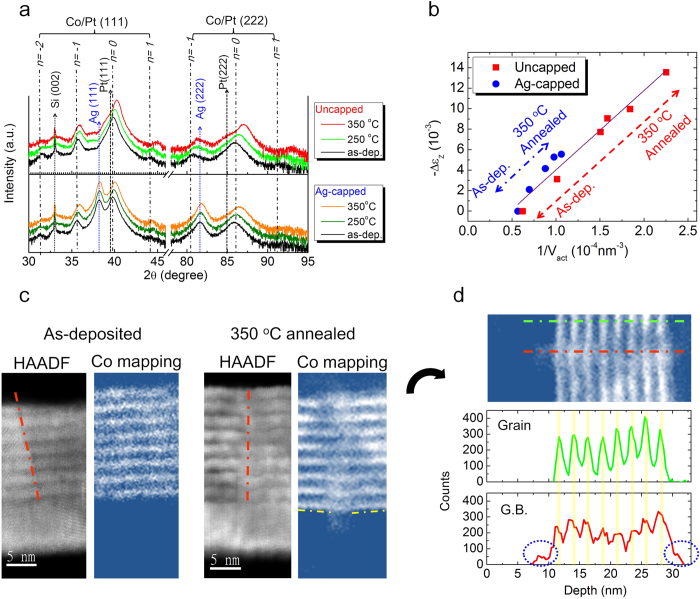
Structural and microstructural analyses of the Co/Pt MLs with different capping conditions (**a**) θ–2θ X-ray diffraction patterns of Co/Pt MLs with different T_ann_. The central (n = 0) and satellite (n = −2, −1, 1) peaks of the uncapped (Ag-capped) samples with increasing T_ann_ are shown in the upper panel (lower panel). (**b**) Dependence between the reciprocal of activation volume (1/V_act_) and the change of strain (−Δε_z_). (**c**) STEM-HAADF images and corresponding STEM-EELS mapping of Co L_2,3_ edge are shown for the as-deposited and 350 ^o^C annealed uncapped samples. The red dashlines on HAADF images indicate the position of grain boundary; The yellow dash-lines on the Co mapping point out the offset of Co layers between adjacent grains. (**d**) Co line scans of the 350^o^C annealed sample extracted from the Co mapping (the same image shown in (**c**), but rotated by 90^o^) on different positions: the green line shows the Co distribution across the whole grain, and the red line is along the grain boundary (as indicated on the Co mapping image). The blue circles on the Co line scan along the grain boundary indicate the distribution of out-diffusion Co via grain boundary creep.

To reveal how intergranular exchange coupling changes with RTA processing and to identify what the role of Ag passivation is, we investigate the structural changes occurred during RTA by performing θ–2θ X-ray diffraction (XRD). Figure 4(a) shows the variations of the central (n = 0) and the satellite (n = −2, −1, 1) XRD peaks of Co/Pt MLs (111) and (222) planes with T_ann_. The central peak position is determined by the average d-spacing of Co/Pt MLs, and the satellite peak intensity reflects interface sharpness between Co and Pt^38^. All samples show a central peak shift to a higher 2θ angle with elevating T_ann_, accompanying the conserved satellite peak intensity when T_ann_ is below 350 ^o^C. The conserved satellite peak intensities indicate a conserved layered structure; therefore, the observed central peak shift should not be related to the bulk diffusion between layers, for example, intermixing or alloying, which would destroy the layered structure. On the other hand, by wafer curvature measurements^39^, we confirm that stress relaxation occurred after RTA process. The as-deposited samples reveal the presence of the in-plane bi-axial compressive stress but the annealed samples show less compressive stress. (see Supplementary Table 1 for details, and Supplementary Note 2 to exclude the magnetostriction effect) Consequently, the central peak shift is attributed to the stress relaxation during RTA processing. Furthermore, we notice that the samples showing larger 2θ shift always accompany higher H_n_ and smaller V_act_ after RTA. To quantitatively analyze the correlation between the stress relaxation in Co/Pt MLs and the corresponding magnetic property changes, we calculate the changes of the strain along the film normal direction (Δε_z_, defined as the strain difference between the as-deposited and the annealed states) based on the central peak shift in XRD. As shown in Fig. 4(b), by plotting the −Δε_z_ (ε_z_ is reduced after annealing) versus the corresponding 1/V_act_, we observe a linear dependence between −Δε_z_ and 1/V_act_, as observed in the H_n_ vs. 1/V_act_, implying the stress relaxation process may strongly correlate with the intergranular exchange coupling of Co/Pt MLs.

To clarify the correlation between stress relaxation and intergranular exchange coupling, we performed cross-sectional imaging with high-angle annular dark field (HAADF) as well as electron energy loss spectra (EELS) of Co L_2,3_-edge mapping by using scanning transmission electron microscope (STEM). As shown in Fig. 4(c), the columnar grains and layered structure of Co/Pt MLs are clearly shown in the STEM-HAADF images for the as-deposited (left) and the 350 ^o^C annealed samples (right). The different contrast of the adjacent grains originates from different crystal orientations of grains with respect to the electron beam direction and the brighter contrast inside each grain represents Pt layers with higher atomic number compared to the darker Co layers. Based on the contrast between grains and the lattice images, we can distinguish the grain boundaries, marked by red dash lines on the HAADF images. We subsequently performed STEM-EELS Co L_2,3_-edge mapping on the same region, where the bright contrast represents the Co distribution. For the as-deposited sample, the Co layers are continuous between the adjacent grains, i.e. across the corresponding grain boundary. On the other hand, for the 350 ^o^C annealed sample, although the Co layers are still distinguishable and continuous inside each grain, the Co layered structure is out-of-sync at grain boundaries, offsetting to each other across grain boundaries, as marked by the yellow dash-line in the 350 ^o^C annealed Co mapping image. As shown in Fig. 4(d), we also extract the line scan data through the grain (shown by the green dash-line) and along the grain boundary (the red dash-line) from the Co L_2,3_-edge mapping of 350 ^o^C annealed sample. Based on the Co L_2,3_-edge EELS signal difference between the layers, we found that the Co layered structure after 350 ^o^C RTA keeps clear periodic distribution of chemical composition throughout the grain, but becomes blurred along the grain boundary. That is, the EELS signal maximums are lower and the distributions of Co along the film normal are wider at grain boundary (red) compared to the case through the grain (green). Moreover, based on the line scan along the grain boundary, we also observed that some Co diffused out of the layered structure along grain boundary, circled by blue in Fig. 4(d), which was not found in the as-deposited samples. (see Supplementary Figure 3 for more details)

To understand the modification of microstructure at grain boundaries and its corresponding changes of the magnetic properties, the stress relaxation process during RTA will be discussed first. Because of the deposition stress during sputtering^40^ (revealed in wafer curvature measurements of the as-deposited sample), and the thermal stress^41^ during RTA, an in-plane bi-axial compressive stress exists in Co/Pt MLs. When temperature exceeds a critical value, diffusion is activated to rearrange the atoms to release the accumulated stress, the so-called diffusion creep, which has been believed as one of the mechanisms governing the stress relaxation in metal films when temperature is raised^42^. Because the layered structure inside grains is conserved but becomes blurred for the chemical composition at grain boundary, we propose that the stress relaxation during RTA is mainly due to diffusion creep via grain boundary diffusion, i.e. the so-called Coble creep^43^. (see Supplementary Figure 4 for the possibility of stress relaxation via grain growth) That is, an out-diffusion mass flow via grain boundaries occurs during RTA processing, including both Co and Pt atoms, which accompanies stress relaxation. Both the out-diffusion Co along grain boundaries and the chemical composition blurred grain boundaries as observed in the EELS Co mapping image are the evidences for the Coble creep mechanism for stress relaxation. Meanwhile, because the out diffusion mass flow along grain boundary includes both Co and Pt atoms, the composition of grain boundaries can be disturbed and the continuity of Co across grain boundaries can also be broken, bringing about Co deficient grain boundaries, where the direct exchange coupling between Co atoms was interrupted by additional Pt atoms, resulting in reduced intergranular exchange coupling^44^. Besides, the out-of-sync grain boundaries with offset Co layers may also decrease the intergranular exchange coupling by reducing the direct Co-Co exchange coupling between the adjacent grains^29^,^44^. Consequently, the larger −Δε_z_ occurred in Co/Pt MLs after RTA process indicates that larger amount of mass flow has been driven to modify grain boundaries, leading to a lower intergranular exchange coupling strength and reduced V_act_ in Co/Pt MLs.

In addition, it has been reported (mostly in BEOL process) that when the stress relaxation is dominated by diffusion creep, an additional passivation layer, possessing good adhesion and low interdiffusivity with the adjacent metal film, can significantly hinder the stress relaxation of metal films by changing its diffusion kinetics^41^,^45^. That is, the free surface of the metal film with a lower energy barrier for diffusion is replaced by the interface between the passivation layer and the metal film with a higher energy barrier. The energy barrier for diffusion raises significantly and impedes the diffusion of atoms or vacancies, and therefore hinders the diffusion creep. Thus, a proper passivation layer suppresses the stress relaxation during annealing, for example the AlO_x_ on Al^46^ and the SiN_x_ on Cu^41^. In our case, the Ag capping layer has been reported to offer a robust Ag/Pt interface with good adhesion and only monolayer interdiffusion^47^ so the Ag capping layer is selected to suppress the stress relaxation. We confirm that no significant compositional profile changes of Ag and Pt occur during RTA (see Supplementary Figure 5). We also exclude the possibility that Ag diffusion modifies magnetic properties (see Supplementary Note 3). As shown in Fig. 4(a), under the same T_ann_, the 2θ shift of the uncapped samples is always larger than those of the Ag-capped samples, implying that an extra Ag capping layer indeed suppresses the stress relaxation during RTA. With a Ag capping layer, the fast free surface diffusion is blocked by the robust Ag/Pt interface and the grain boundary diffusion is therefore retarded, leading to less stress relaxation and the less amount of modified grain boundaries. Furthermore, we found that the trend of −Δε_z_ vs. 1/V_act_ lies in the same line for both the uncapped and Ag-capped samples, shown in Fig. 4(b), indicating that the intergranular exchange coupling of Co/Pt MLs is strongly related to the degree of stress relaxation, which can be altered by either RTA temperature or passivation layer. This concept can be further verified by using different passivation layers. For example, Ta is another good passivation layer due to good adhesion between Ta and Pt, but SiO_2_ is not (see Supplementary Figure 6 for details). Simply by selecting proper pre-patterned passivation layers, we can alter the diffusion kinetics and laterally modify the intergranular exchange coupling to achieve the proposed magnetic patterning.

### Magnetic patterning by local Joule heating

**Figure 5:**
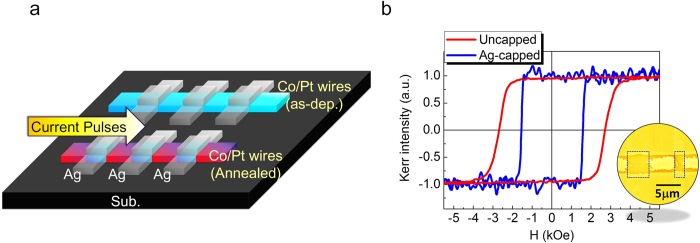
Magnetic patterning by Joule heating (**a**) A sketch of the process flow for Joule heating annealing, where individual wire can be selected for magnetic patterning. (**b)** Hysteresis loops obtained by FMOKE at different regions on the magnetic patterned wire. The insertion of (**b**) shows the MFM image of magnetic patterned magnetic wire at the partially saturated states. The Ag-capped region is indicated by blue dot-squares with different width of Ag pre-pattern (4 and 2 μm, respectively).

As discussed in previous paragraphs, the magnetic properties can be locally manipulated by controlling stress relaxation during annealing process. However, the infared lamp used in our RTA process, providing uniform heating area for the whole sample, should not be the only driving force for stress relaxation. Here we demonstrate how Joule heating^48^ generated by currents can replace the RTA process so that we can selectively anneal the designated magnetic wires. The Joule heating method provides another route to manipulate the spatial distribution of magnetic properties. As shown in Fig. 5(a), by applying current pulses (40 pulses with the current density of 45MA/cm^2^ and the duration of 50 ms for each pulse) into the selected magnetic wires, only the wires with current are annealed. The same passivation effects as RTA are observed by using Joule heating annealing. As shown in Fig. 5(b), the hysteresis loops acquired by FMOKE reveal that the magnetization switching of the Ag-capped regions occurs at a lower field than that of the uncapped regions. Inset of Fig. 5(b) shows the partially reversed MFM images of the magnetic patterned Co/Pt MLs wires by Joule heating annealing. Indeed, the reversed domain first nucleated in Ag-capped regions (enclosed by blue dot-squares) and the domain walls was pinned at the boundary between the Ag-capped and uncapped regions, that is, the magnetic patterning can be achieved not only by RTA but by Joule heating annealing.

### Uniqueness of proposed magnetic patterning

To diversify the magnetic patterning methods is of great importance for designing new devices and extending their functionality. The most unique feature for our magnetic patterning method is to adjust intergranular exchange coupling without significantly changing K_eff_ and M_s_ in all regions, which have not been achieved before and can provide a new degree of freedom to design magnetic devices. The modified intergranular exchange coupling has been shown to alter the H_n_ and H_p_ due to the modified V_act_ and n_pin_, respectively. In current-driven domain wall motion devices, the magnetic patterning has been used for creating the nucleation sites or pinning sites in magnetic wires to control the domain wall motion. Our magnetic patterning method, which alters the H_n_ and H_p_ in local regions, can be used for defining the storage bits in the magnetic wires for the so-called racetrack memory^4^. Besides, we can generate different H_n_ laterally in the magnetic wires. Therefore, the magnonic crystal can be potentially obtained by introducing various H_n_ instead of M_s_ in different area to achieve periodical magnetization states (partially switched state, like the cases in Fig. 1(c) and the insertion of 5(b)).

Compared to ion implantation or other magnetic patterning methods, our approach can achieve a nanoscale magnetic patterning as demonstrated previously and is possible for a large-scale patterning region (across the whole wafer) without special or expensive equipment. Besides, different from ion implantation that may damage the dielectric layers and the logic devices underneath within the VLSI (very-large-scale integration) framework, one of the advantages of our method is BEOL compatible (as discussed in Supplementary Figure 6). Furthermore, by Joule heating annealing, our magnetic patterning cannot only be performed on the selected magnetic wires, but can be potentially carried out after the whole IC packaging process, opening a new route for post-production programing of magnetic devices. Furthermore, because the passivation effect on stress relaxation is a common phenomenon observed in many other metal films, we believe our approach can be further extended to other multilayers structure, for instance Co/Pd^49^, Co/Ni^50^ multilayers, or even other kinds of perpendicular magnetic anisotropy materials. By properly choosing a passivation material, we can achieve magnetic patterning in other systems.

Regarding the resolution of our magnetic patterning, our proposed method is mainly confined by the roughness of the domain wall. The domain wall is defined by the edges of Ag wires because the regions with Ag capping show small H_n_ and H_p_. Since we used a lift-off process to define the Ag lines, we may introduce artificial defects at edges and thus induce the domain wall roughness. If we optimize the lift-off process or use the etching process to well define the edges of Ag wires, the resolution of our proposed method can be further improved.

## Conclusions

We have demonstrated a new magnetic patterning method by adopting pre-patterned passivation layers, Ag, with thermal treatments, including RTA and Joule heating annealing. The working principle of our proposed magnetic patterning relies on the control of stress relaxation in Co/Pt MLs during thermal treatment. The modification of grain boundaries occurs during thermal treatments due to Coble creep, leading to the changes of intergranular exchange coupling. The pre-patterned Ag passivation layer is shown to control the degree of stress relaxation and thus the strength of intergranular exchange coupling. When the intergranular exchange coupling is altered, the H_n_, H_p_, and D_domain_ are modified accordingly; therefore, the magnetic properties in the regions can be engineered depending on with Ag or without Ag capping, leading to lateral distribution of magnetic properties. Our proposed approach diversifies the magnetic patterning methods, that is, changing H_n_ and H_p_ without altering M_s_ or K_eff_, which may offer a new degree of freedom for designing novel magnetic devices.

## Methods

We prepared the Co/Pt MLs by a ultra-high vacuum magnetron sputtering system with the base pressure about 4 × 10^−8^ Torr. The multilayers structure we used in this work is substrate/Ta(3 nm)/Pt(7 nm)/[Co(0.4 nm)/Pt(2 nm)]_8_ deposited on thermally oxidized Si (100) substrates. A 20 nm thick Ag passivation layer was then deposited on some of the multilayers. The working pressure of Ar was 7 mTorr for Pt, and 3 mTorr for the others. Rapid thermal annealing with infrared lamps was subsequently performed at temperatures from 200 ^o^C to 350 ^o^C with a duration of 20 seconds and a ramp rate of 25 ^o^C/sec under the vacuum of 5 × 10^−5^ Torr.

To demonstrate the magnetic patterning, we first pre-patterned the Ag capping layer with different stripes width by e-beam lithography and lift-off process. After the Ag pattern was prepared, RTA was carried out. After RTA, the Ag stripes were selectively removed by NH_4_OH(30wt%):H_2_O_2_(30wt%) etching solution, which effectively etches Ag away without reacting with the MLs. For the magnetic patterning on a single magnetic wire by Joule heating annealing, we use conventional photolithography and ICP-RIE (inductively coupled plasma reactive ion etching) for defining wires of Co/Pt MLs; the Ag pre-patterns are subsequently defined by photolithography and lift-off. By controlling the amplitude of current density, the numbers of pulses, and the duration for each pulse, we can modify magnetic properties. Here, we use 40 pulses with the current density of 45MA/cm^2^ and the duration of 50 ms for each pulse. By *in-situ* monitoring the resistance change with external magnetic fields, that is, measuring magnon magnetoresistance^51^, we can obtain the coercivity so that we can adjust the current pulses and durations accordingly to modify the magnetic properties.

A vibration sample magnetometer (VSM) was used for in-plane and out-of-plane hysteresis loops of the Co/Pt MLs. H_n_, M_S_, and H_p_ were extracted from the out-of-plane hysteresis loops with initial curves, where H_n_ is defined by the onset of magnetization drop from saturated state in the hysteresis loops, and H_p_ is defined by the first slope change of initial curves; H_K_ was estimated from the saturation field of in-plane loops. VSM was used to measure magnetization relaxation curves for calculating the effective activation volume and to acquire ΔM curves. The magnetic force microscopy (MFM) and corresponding atomic force microscopy (AFM) were carried out for estimating the ac-demagnetized domain size and revealing magnetic patterns in local regions. The ac-demagnetized domain size (D_domain_) was quantitatively estimated by using two dimensional fast Fourier transformations (2D-FFT) technique for each corresponding MFM image. The focused magneto-optical Kerr effect measurement with 2 μm laser spot was performed for the hysteresis loops in local regions. The wafer curvature measurement was carried out for stress analyses in Co/Pt MLs. The θ–2θ X-ray diffraction (XRD) was performed for measuring the change of strain of Co/Pt MLs before and after RTA process. The cross-sectional high angle annular dark field images and corresponding element mapping were acquired by Cs-corrected scanning transmission electron microscope (STEM) and electron energy loss spectra (EELS), respectively, with the electron microscope operating at 200 kV.

## Additional Information

**How to cite this article**: Huang, K.-F. *et al.* Magnetic patterning: local manipulation of the intergranular exchange coupling via grain boundary engineering. *Sci. Rep.* **5**, 11904; doi: 10.1038/srep11904 (2015).

## Supplementary Material

Supplementary Information
