# Supplementary material for: Magnetic patterning: local manipulation of the intergranular exchange coupling via grain boundary engineering
Source: Sci Rep. 2015 Jul 9;5:11904. doi: 10.1038/srep11904 (PMC4496669; doi:10.1038/srep11904)
Supplement: Supplementary Information [file srep11904-s1.pdf]

Magnetic patterning: local manipulation of the intergranular exchange coupling via grain boundary engineering

Kuo-Feng Huang,[1] Jung-Wei Liao,[1] Cheng-Yu Hsieh,[2] Liang-Wei Wang,[1] Yen-Chun Huang,[1] Wei-Chih Wen,[1] Mu-Tung Chang,[2] Shen-Chuan Lo,[2] Jun Yuan,[3] Hsiu-Hau Lin,[4] and Chih-Huang Lai[1]*

[1]Department of Materials Science and Engineering, National Tsing Hua University, Hsinchu, 300, Taiwan

[2]Material and Chemical Research Laboratories, Nanotechnology Research Center, Industrial Technology Research Institute, Hsinchu, 310, Taiwan

[3]Department of Physics, University of York, Heslington, York, YO10 5DD, United Kingdom

[4]Department of Physics, National Tsing Hua University, Hsinchu, 300, Taiwan

* Correspondence and requests for materials should be addressed to C.-H. L. (chlai@mx.nthu.edu.tw)

Supplementary Figures

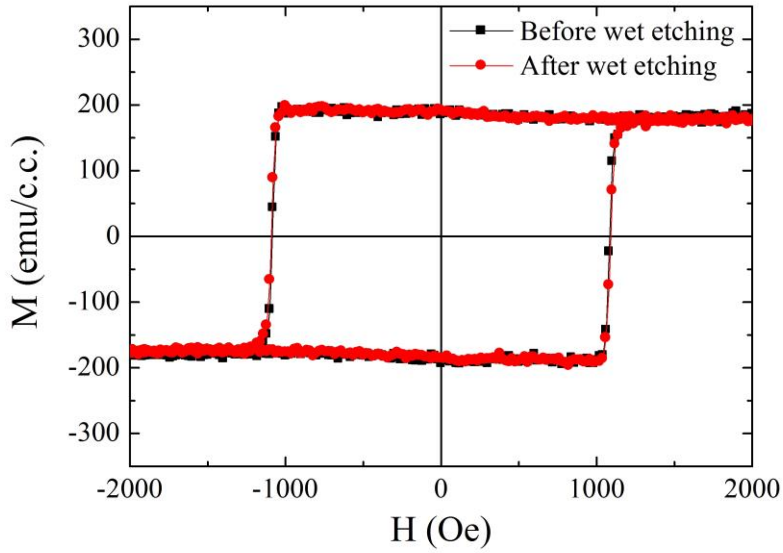

Supplementary Figure 1. Effects of Ag removal on magnetic properties. Hysteresis loops before and after NH_4OH:H_2O_2 wet etching process are shown. The hysteresis loop of Co/Pt MLs is not changed by the wet etching process of the Ag removal, indicating no damage on Co/Pt MLs after removing the Ag capping layer.

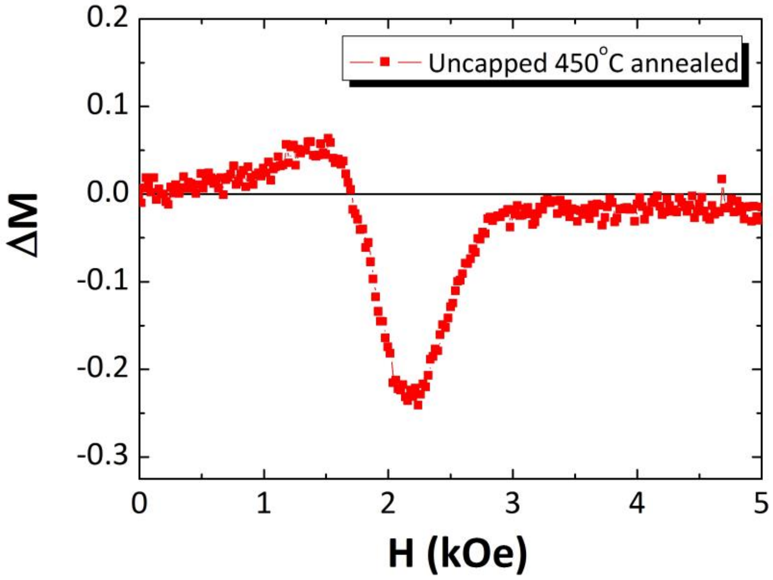

Supplementary Figure 2. Magnetic properties of Co/Pt MLs when RTA temperature is higher than 350°C. In the article, we discuss the properties for the samples annealed at or below 350℃, which keeps the magnetization reversal dominated by intergranular exchange coupling. When T_{ann} is increased above 350° C, much serious diffusion may occur, leading to changes of multilayer structure and magnetization reversal. **Supplementary Figure 2** reveals that the ΔM peak becomes negative after 450℃ annealing, indicating a dipolar interaction dominated magnetization reversal, different from that of samples processed with the low RTA temperature. Consequently, the evolution of magnetic properties with annealing temperature may not follow the same trend as the samples annealed below 350℃.

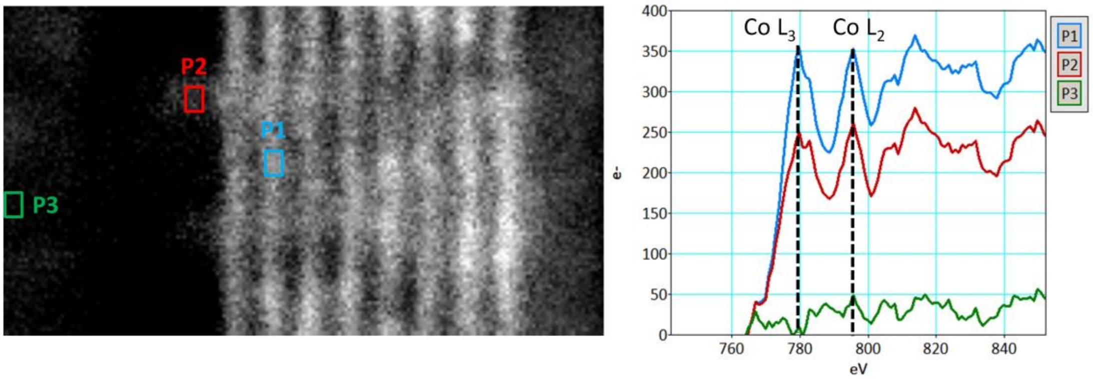

Supplementary Figure 3. EELS spectra of the out-diffusion Co atoms. In the article, based on the EELS mapping on Co $L_{2,3}$-edge, we have claimed that the main Co diffusion is along grain boundary. Here, we extract the EELS spectra for confirming if the contrast comes from Co. As shown in the left of **Supplementary**

Figure 3, we choose 3 different positions, where P1 is the layered Co, P2 is the out-diffusion Co, P3 is the substrate. Based on the EELS spectra as shown in the right of **Supplementary Figure 3**, we observed Co absorption edge on P1 and P2, indicating the bright contrast of these two positions are from the existence of Co atoms. For P3, no Co absorption edge was found from the EELS spectra, implying the weak bright contrast should come from noise. That is, based on the existence of Co absorption edge on EELS spectra, we have confirmed that the EELS mapping supporting on Co diffusion along grain boundary is reliable.

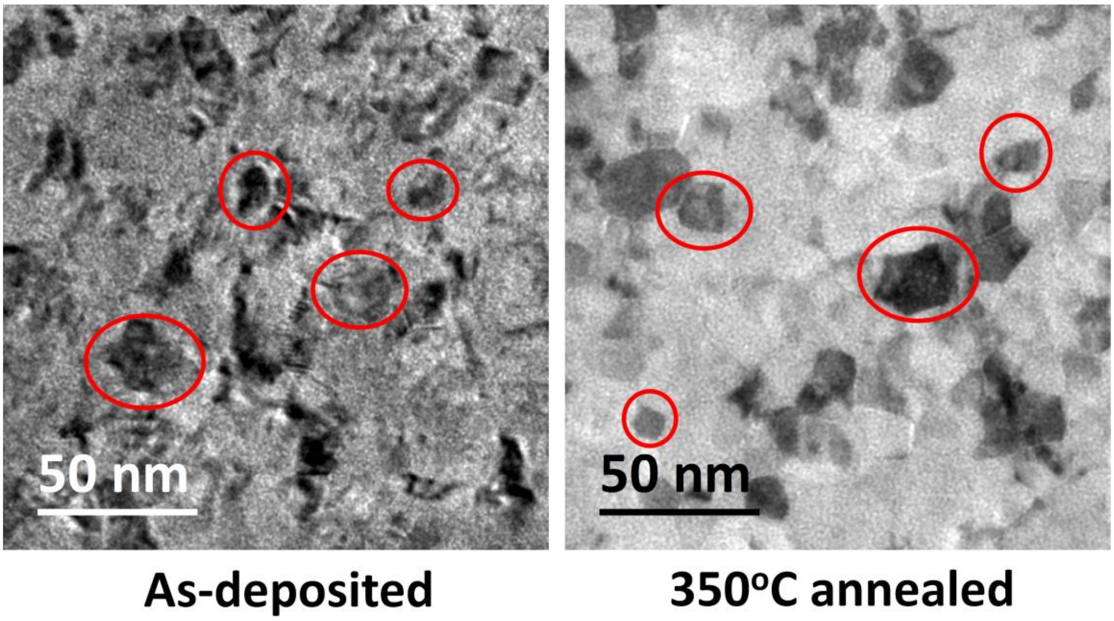

Supplementary Figure 4. Exclude the possibility of stress relaxation via grain growth. To evaluate the possibility of stress relaxation via grain growth, we took plan-view bright field TEM images for grain size distribution before and after RTA. As shown in the plan-view bright field TEM images, shown in **Supplementary**

Figure 4, the mean grain sizes of the as-deposited and annealed samples from 50 distinguishable grains, circled in **Supplementary Figure 4** as examples, are 14.0 ± 3.0nm and 13.8 ± 3.9nm, respectively: no notable grain growth was found after 350°C RTA. Consequently, the grain growth should not be responsible for the stress relaxation.

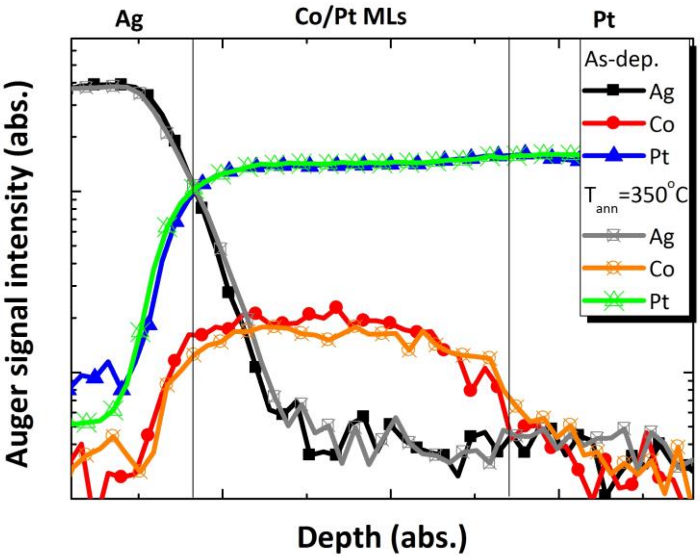

Supplementary Figure 5. Auger depth profiles before and after RTA. By Auger depth profiles, as shown in **Supplementary Figure 5**, we also found no significant compositional profile changes of Ag and Pt occur during RTA. Our results are also consistent with the reference we cited in the article that Ag/Pt interface is robust for preventing interdiffusion.[1]

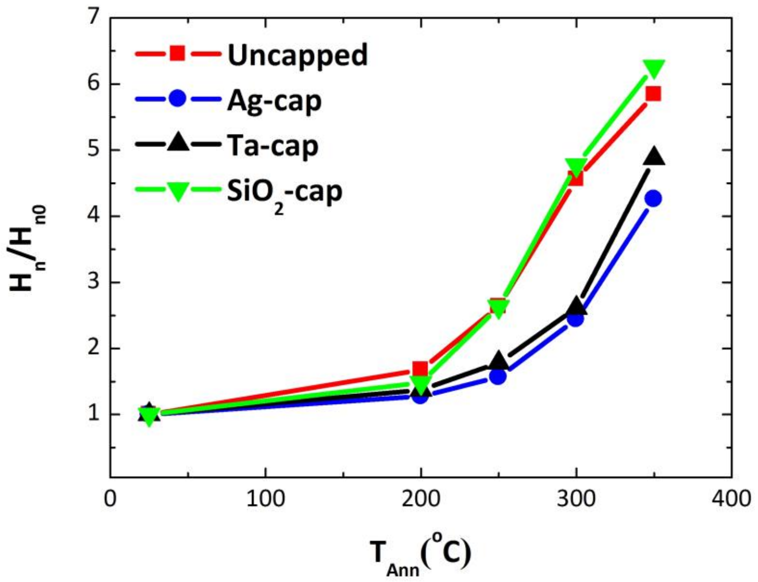

Supplementary Figure 6. Validation of the passivation layer effect for diffusion creep mechanism by using Ta and SiO_2. To reconfirm the diffusion creep mechanism, we selected two other capping layers: Ta (with good adhesion to Pt)[2] and SiO_2 (with poor adhesion to Pt) on Co/Pt MLs and carried out RTA process. As shown in **Supplementary Figure 6**, the annealing temperature dependence of H_n with Ta-capped samples is much similar to the Ag-capped ones, but the dependence for SiO_2-capped sample is more like the uncapped case. Based on diffusion creep, the good adhesion between Ta and Pt provides a stable interface, suppressing out-diffusion atoms and blocking the diffusion paths along grain boundaries, as the Ag/Pt interface does. In contrast, the adhesion between SiO_2 and Pt has long been known very poor due to the inferior affinity between noble metal to oxygen.[3] Although the Co/Pt MLs is fully covered by SiO_2, the SiO_2/Pt interface with poor

adhesion may still behave as an out-diffusion atom sink. Consequently, the grain boundary diffusion cannot be impeded by SiO_2 passivation layer with poor adhesion, which is similar to the case without capping layers. Therefore, both the Ta-capped and SiO_2-capped cases can be explained by the diffusion creep to support the mechanism we proposed for modification of magnetic properties.

Furthermore, because different capping layer may contribute to different degree of stress relaxation and magnetic properties, the magnetic patterning can be achieved by choosing proper combination of capping materials, for example, Ta and SiO_2, which are both widely used in BEOL process. Therefore, by pre-pattern Ta and SiO_2 capping layer on magnetic devices, the magnetic patterning can therefore achieve by Joule heating annealing even after the magnetic devices are sealed by IC packaging.

Supplementary Table

Samples	blank substrate [m^{-1}]	As-deposited [m^{-1}]	T_{ann}=350℃ [m^{-1}]
Uncapped	-0.02305	-0.08255	0.00311
Ag-capped	-0.03852	-0.11353	-0.02594

Supplementary Table 1. Stress state of the Co/Pt MLs obtained by curvature measurement. Wafer curvature of each stage for the uncapped and Ag-capped samples are listed (The substrate is Si (001) single crystal with 125 μm in thickness).

The wafer curvature measurement can provide us the stress state of the whole structure, but not the exact stress inside Co/Pt MLs. **Supplementary Table 1** shows the sample curvature of the Co/Pt MLs at different stages from the blank substrate to that after 350℃ RTA processing. The high negative curvature value of the as-deposited state shows that the whole sample is under a compressive stress. After annealing, the absolute value of curvature reduces or even becomes a positive value, indicating that the compressive stress is released after RTA process. However, to quantitatively analyze the stress change in Co/Pt MLs only, we need Young's modulus of the layers and complicated modeling to account for the multilayer structure. Therefore, we use XRD spectra to reveal the strain change ($\Delta\varepsilon_z$) to show the stress relaxation.

Supplementary Notes

Supplementary Note 1.

Explanation for the dependence between $1/V_{act}$ and H_n by curling mode of nucleation process. The linear dependence between $1/V_{act}$ and H_n, as shown in Figure 3(e), can be quantitatively described by using the curling mode for nucleation.[4] The curling mode is a mode of nucleation with a plane of curling spins in nuclei. Based on this curling mode, H_n can be expressed by the following equation,[4]

$$H_n = \frac{2K}{M_s} - DM_s + \frac{c(D)A}{M_s R^2}$$ (**Supplementary Equation 1**)

where K is the crystal anisotropy energy, D is the demagnetizing factor, c(D) is a constant determined by the different shape of the nuclei and the type of the curled spin structure, A is the exchange stiffness, and R is the radius of the curling plane. Because the Co/Pt MLs in our case is a sheet film with perpendicular magnetic anisotropy, we presume that the curling plane should be in the plane of the Co/Pt ML film; therefore, the V_{act} for nucleation based on the curling mode can be estimated by using a planar volume of $\pi R^2 d$, where d is the thickness of the Co/Pt MLs. Therefore, the **Supplementary Equation 1** can be rewritten as

$$H_n = H_{n0} + \left(\frac{c(D)A\pi d}{M_s}\right)\frac{1}{V_{act}}$$ (**Supplementary Equation 2**)

where H_{n0} ($= \frac{2K}{M_s} - DM_s$) is the V_{act} independent part. This equation suggests that the nucleation field increases inversely with V_{act}. Based on the curling mode, the formation of the curled spin structure becomes more difficult when V_{act} is reduced due to the increased exchange energy. Therefore, the nucleation energy barrier, determining H_n, also increases with a reduction of V_{act} as the **Supplementary Equation 2** described.

Supplementary Note 2.

Evaluation on the contribution of magnetostriction effect. Since the stress state is changed after RTA, we would like to evaluate the contribution of magnetostriction to the change of K_{eff} and H_n. We extract the largest observed change of stress (~13×10^8 dyne/cm^2) from wafer curvature together with the magnetostriction coefficient (λ) of Co/Pt (3×10^{-5}).[5] The estimated K_{eff} is -6×10^4erg/c.c., which is relative small and opposite to our observed enhanced H_n after RTA. This result confirms that the change of H_n is not due to the change of anisotropy from magnetostriction.

Supplementary Note 3.

Discussion on the posibility of magnetic properties modified by Ag diffusion. Here we discuss the possibility that the observed magnetic property changes originate from Ag diffusing into Co/Pt MLs. If the bulk diffusion of Ag into Co/Pt MLs occurs, the multilayer structure may be destroyed by Ag mass flow, accompanying the reduction of K_{eff}. However, both the satellite peak intensity and K_{eff} are almost identical for uncapped and Ag-capped samples with the same T_{ann}. If grain boundary diffusion takes place, the additional Ag atoms at grain boundary may block the Co-Co direct exchange coupling between grains and lead to smaller V_{act} with higher H_n compared to uncapped cases.[6] However, we found a larger V_{act} and smaller H_n for Ag-capped samples than those of the corresponding uncapped ones, which is contrary

to the assumption for the Ag diffusion along grain boundaries. Therefore, we can exclude the possibility that Ag diffusion leads to the observed changes of magnetic properties. Our results are also consistent with the reference we cited in the article that Ag/Pt interface is robust for preventing interdiffusion.[1]

Supplementary References:

1. Roder H, Schuster R, Brune H, Kern K. Monolayer-Confined Mixing at the Ag-Pt(111) Interface. *Phys Rev Lett* **71**, 2086-2089 (1993).

2. Firebaugh SL, Jensen KF, Schmidt MA. Investigation of high-temperature degradation of platinum thin films with an in situ resistance measurement apparatus. *J Microelectromech S* **7**, 128-135 (1998).

3. Gan DW, *et al.* Isothermal stress relaxation in electroplated Cu films. I. Mass transport measurements. *J Appl Phys* **97**, 103531 (2005).

4. Skomski R. Nanomagnetics. *J Phys-Condes Matter* **15**, R841-R896 (2003).

5. Hashimoto S, Ochiai Y, Aso K. Perpendicular Magnetic-Anisotropy and Magnetostriction of Sputtered Co/Pd and Co/Pt Multilayered Films. *J Appl Phys* **66**, 4909-4916 (1989).

6. Zhao ZL, Ding J, Inaba K, Chen JS, Wang JP. Promotion of L1(0) ordered phase transformation by the Ag top layer on FePt thin films. *Appl Phys Lett* **83**, 2196-2198 (2003).
